# Polymorphism of Folate Metabolism Genes among Ethnic Kazakh Women with Preeclampsia in Kazakhstan: A Descriptive Study

**DOI:** 10.3390/biology13090648

**Published:** 2024-08-23

**Authors:** Lyazzat Kaldygulova, Sauran Yerdessov, Talshyn Ukybassova, Yevgeniy Kim, Dinmukhamed Ayaganov, Andrey Gaiday

**Affiliations:** 1 Department of Obstetrics and Gynecology #2, West-Kazakhstan Marat Ospanov Medical University, Aktobe 030012, Kazakhstan; a.gaiday@mail.ru; 2 Department of Medicine, School of Medicine, Nazarbayev University, Astana 010000, Kazakhstan; sauran.yerdessov@nu.edu.kz; 3 Clinical Academic Department of Women’s Health, CF “University Medical Center”, Astana 010000, Kazakhstan; talshynu@yandex.ru (T.U.); evg.kim94@gmail.com (Y.K.); 4 LLP “In Vitro” Laboratory, Astana 010000, Kazakhstan; 5 Department of Neurology, Psychiatry, and Narcology, West-Kazakhstan Marat Ospanov Medical University, Aktobe 030012, Kazakhstan; dimash.83@mail.ru

**Keywords:** folate metabolism, methionine synthase, methionine synthase reductase, methylenetetrahydrofolate reductase, MTR, MTRR, MTHFR, pregnancy, preeclampsia, thrombophilia

## Abstract

**Simple Summary:**

Preeclampsia is a severe complication of pregnancy with no clear etiology but confirmed to have complex pathogenetic mechanisms involved. Previous research evidence reported associations between folate metabolism genes’ polymorphisms and preeclampsia. This study aimed to evaluate the prevalence of methionine synthase (*MTR*), methionine synthase reductase (*MTRR)*, and methylenetetrahydrofolate reductase (*MTHFR*) genes’ polymorphisms among ethnic Kazakh women with preeclampsia. The results of this study provide a basic understanding of folate metabolism genes’ polymorphisms in preeclampsia among the Central Asian population.

**Abstract:**

Introduction: Preeclampsia is a severe multifactorial complication of pregnancy. Studies found associations between folate metabolism genes’ polymorphisms and preeclampsia. However, investigations in this field are limited among Asian populations. Thus, the study’s aim was to evaluate the prevalence of methionine synthase (*MTR*), methionine synthase reductase (*MTRR)*, and methylenetetrahydrofolate reductase (*MTHFR*) genes’ polymorphisms among ethnic Kazakh women with preeclampsia. Methods: This was a retrospective study involving 4246 patients’ data for the period of 2018–2022. Identification of *MTR*, *MTRR*, and *MTHFR* genes’ polymorphism was performed via PR-PCR. Peripheral blood samples were obtained for the analyses. In total, 4246 patients’ data of Kazakh ethnicity with preeclampsia at >20 weeks gestational age who had undergone an investigation to identify polymorphisms of the folate metabolism pathway genes for the period of 5 years were included in this study. Results: The most common and prevalent mutation was the *MTRR A66G* polymorphism: 24.5% of all tested patients with preeclampsia had the *MTRR A66G* polymorphism. It was highest among the 35–39 age group participants. The second most prevalent was the *MTHFR C677T* polymorphism: 9% of women with preeclampsia had the *MTHFR C677T* mutation. It was highest among women aged 30–34. There was a rare association of the *MTR A2756G* mutation with preeclampsia among the study participants. Conclusions: The identified levels of *MTRR A66G* and *MTHFR C677T* polymorphisms among the study participants suggest the importance of evaluating *MTRR* and *MTHFR* polymorphisms in women with preeclampsia. The role of the *MTR A2756G* polymorphism in the development of preeclampsia needs to be further investigated.

## 1. Introduction

Preeclampsia is a complication of pregnancy characterized by proteinuria and hypertensive aberrations [1,2,3,4,5]. Preeclampsia usually occurs after 20 weeks of gestation and is characterized by elevation of blood pressure (systolic ≥140 mm Hg and diastolic ≥90 mm Hg) and proteinuria of ≥300 mg per 24 h [2].

Preeclampsia complicates up to 8% of pregnancies worldwide, and for many decades, it has remained one of the major causes of fetal and maternal mortality [1,4,6,7]. Moreover, the prevalence of preeclampsia differs among races and ethnic groups by 7–11% [5,8]. Although the primary etiology of preeclampsia remains unknown, some important steps in the pathogenesis of preeclampsia have been elucidated as an abnormal trophoblastic invasion followed by uteroplacental hypoperfusion [3,4,5,9].

During pregnancy, the overall body homeostasis is affected by changes in the majority of the organ systems. The changes in the cardiovascular system, blood volume, and liver function result in pregnancy being a hypercoagulation condition [10,11,12]. Various types of thrombophilia are accepted as risk factors for preeclampsia [13,14,15,16,17]. Thus, pregnancy-related hypercoagulation status in pregnant women with hereditary thrombophilia is at a higher risk of preeclampsia [15,16,18]. The most prevalent hereditary types of thrombophilia associated with preeclampsia are factor V Leiden mutation and genetic polymorphism of folate metabolism pathway-related genes (methionine synthase reductase (MTRR) and methylenetetrahydrofolate reductase (MTHFR) enzymes) [15,16,19,20,21]. 

Methionine synthase (MTR), MTRR, and MTHFR are the key enzymes in folate and homocysteine metabolism pathways [5,22,23]. Polymorphisms of these genes responsible for folate pathway enzymes’ function contribute to hyperhomocysteinemia, which is proven to cause endothelial injury through increased oxidative stress [10,16,20]. These pathologic changes later lead to aberrations in the endothelial synthesis of vasodilatory agents and increase the synthesis coagulation factors and platelet aggregation [5,10,21,23]. 

Mutations in *MTRR* and *MTHFR* genes cause changes in the gene expression and function of the MTRR enzyme and affect the level of homocysteine in the plasma leading to vascular endothelial damage and dysfunction leading to thrombophilia [23,24], which is an important factor in increasing the risk of preeclampsia [5,25,26]. However, the role of the *MTR* gene polymorphism in the pathogenesis of preeclampsia is not clearly determined [25]. MTHFR is a vital enzyme for folic acid metabolism, which is an essential vitamin for conception and pregnancy maintenance (endometrial receptivity, oocyte development, embryo implantation, and pregnancy development) [5,22,27]. Low levels of folate and high levels of homocysteine are found among women with preeclampsia; thus, this fact proves an essential fore of MTR, MTRR, and MTHFR enzymes in coagulation homeostasis and pregnancy maintenance [5,22,23,25,26]. Moreover, multiple studies found that genetic polymorphisms, particularly *MTR A2756G*, *MTRR A66Gm*, and *MTHFR C677T*, are associated with preeclampsia and severe preeclampsia [13,14,25,26,28]. *MTHFR C677T* polymorphism, together with an associated folic acid, cyanocobalamin, and pyridoxine deficiency, could lead to hyperhomocysteinemia, which in turn might cause endothelial dysfunction and activation of the blood coagulation [4,5]. 

The Republic of Kazakhstan is a middle-income country located in Central Asia. The country’s population is 19.6 million, with reproductive-aged women accounting for around 26% of the general population [29,30,31]. The Kazakhstani population is represented by many different ethnicities. The title ethnic group of the country is the ethnic Kazakhs (63.1%), followed by ethnic Russians (23.7%), Uzbeks (2.9%), Ukrainians (2.1%), Uygurs (1.4%), Tatars (1.3%), and Germans (1.1%) [32,33]. Kazakh ethnicity belongs to the Turkic ethno-cultural group with a specific fertility cult, which is based on the tradition of having many children [32]. Thus, women’s ability to conceive and give birth to offspring is a cornerstone of family formation and has specific social and economic reasoning behind it [30,32,34,35]. 

The prevalence of the folate genes’ mutations varies among different ethnic groups, with a known high prevalence of the *MTHFR C677T* polymorphisms in the Southern European population and lower levels among African Americans and Germans [5,36]. Very limited data are available about the mutations in the folate metabolism genes in the Asian population [37,38]. The high prevalence is reported among the Japanese population, which is up to 15% of the population [36,39]. Moreover, the risk of the *MTHFR C677T* polymorphism is associated with an increased risk for preeclampsia, especially among the Asian population [18]. In Kazakhstan, low folate status and mild homocysteinemia are reported among the Kazakh population due to their traditional diet with high meat consumption and low vegetables and leafy green intake [37,40]. Moreover, researchers found high levels of the *MTHFR C677T* mutation among the Kazakh population [37]. However, no investigations studied MTR and MTRR enzymes among the Kazakhstani population. Over the past decade, multiple molecular epidemiological studies worldwide investigated the association between folate metabolism genes’ mutations in patients with preeclampsia [13,14,25,26,28]. However, the epidemiology of folate metabolism genes’ polymorphisms has never been investigated among ethnic Kazakh women with preeclampsia. Thus, the aim of this study was to evaluate the prevalence of folate metabolism genes’ polymorphisms (*MTR*, *MTRR*, and *MTHFR*) among ethnic Kazakh women with preeclampsia in the Kazakhstani population. 

## 2. Materials and Methods

### 2.1. Study Design and Study Subjects

This was a retrospective study involving 4246 patients who have undergone analysis to identify *MTR*, *MTRR*, and *MTHFR* genes’ polymorphism in the “In Vitro” laboratory in Kazakhstan. The following inclusion criteria were followed: patients of female gender belonging to Kazakh ethnicity of reproductive age with a singleton pregnancy and clinical and laboratory evidence of preeclampsia were included in the study. Exclusion criteria: patients of other ethnic groups presented in the country, those with a history of recurrent pregnancy loss, without diagnosed/confirmed preeclampsia, and advanced age at the current pregnancy. For this study, the American College of Obstetricians and Gynecologists (ACOG) definition of preeclampsia was used, and women with hypertension (systolic ≥140 mm Hg and diastolic ≥ 90 mm Hg) and proteinuria (≥300 mg per 24 h) at >20 weeks of pregnancy were included [1,2]. Patients were tested at the time of confirmation of preeclampsia diagnosis (second and third trimester of the current pregnancy). 

### 2.2. Ethical Considerations

The study was conducted according to the Helsinki Declaration on research conducted with human subjects [41]. The study was approved by the Research Ethics Committee of the West-Kazakhstan Medical University on 19 November 2021 (protocol #01-05-07-21-2020). Exemption from informed consent has been granted to this study due to the retrospective nature of the study in which only anonymous data were analyzed. 

### 2.3. Laboratory Setting

The laboratory tests were performed in the “In Vitro” laboratory, which is an internationally accredited laboratory (accreditation certificate ISO 15189-2015) [42] working in seven countries of the Commonwealth of Independent States (CIS). The data analyzed in this study are cumulative results obtained in all branches across Kazakhstan, thus, representing the country.

### 2.4. Genes

Three genes of the folate metabolism pathway, *MTR*, *MTRR*, and *MTHFR*, were investigated in this study. The genes and their thrombophilic mutations, which were considered in this study, are presented in Table 1. 

### 2.5. Blood Collection and Genomic DNA Isolation

For the analysis, after overnight fasting, 5.0 milliliters of peripheral blood samples were obtained from each of the study subjects in EDTA-containing tubes. Genomic DNA was extracted from the cell pellet in whole blood using the Promega Wizard^®^ Genomic DNA Purification Kit following a standard method according to the producers’ instructions. The real-time polymerase chain reaction (RT-PCR) using CFX-96 Real-Time System (Singapore) and Vector Best (Russia) reagents with specific primers (Table 2) for PCR were used to perform the analysis for detection of the *MTR A2756G, MTRR A66G*, and *MTHFR C677T* genotypes. The following RT-PCR cycle parameters were followed: 94 °C for 2 min, then 35 cycles of amplification (94 °C 30 s, 60 °C 30 s, and 72° 30 s). The final elongation step of 10 min, 72 °C, and 5 mL of the reaction product were analyzed in a 1.5% agarose gel. The normal, heterozygous, and homozygous mutant genotype profiles of each of the genes were identified. 

### 2.6. Statistical Analysis

Descriptive statistics were utilized to provide a summary of the frequencies of each gene polymorphism within the study population. Chi-squared (χ^2^) tests were employed to evaluate the relationship between individual polymorphisms and their association with age groups. Genotype frequencies were calculated for each polymorphism within different age groups and years of enrollment, and the Hardy–Weinberg equilibrium (HWE) was tested. All of the statistical analyses were carried out using STATA 16.1 (STATA Corporation, College Station, TX, USA). In all analyses, *p*-values were calculated as two-sided, and statistical significance was defined as *p* < 0.05.

## 3. Results

In total, 4246 patients’ data of Kazakh ethnicity with preeclampsia at >20 weeks gestational age who had undergone an investigation to identify polymorphisms of the folate metabolism pathway genes for the period of 5 years (2018–2022) were included in this study. All patients were tested to identify *MTHFR C677T* mutations; however, only 2140 patients simultaneously had *MTR A2756G* testing, and 3082 had *MTRR A66G* analysis performed (Table 3). 

Patients according to their age were subdivided into age groups according to the World Health Organization (WHO) and Population Pyramid standards [44,45]. Most of the patients with preeclampsia tested for all three gene polymorphisms (*MTR A2756G, MTRR A66G*, and *MTHFR C677T*) belonged to the age group of 30–34—27%, followed by patients of 35–39 age group—22%, and 24–29-year-old patients—20% (Table 3). 

Among all folate metabolism genes, the most common and prevalent polymorphism was identified for the *MTRR* gene. From 3082 patients with preeclampsia tested for *MTRR* gene mutations, 756 (24.5%) had confirmed *MTRR A66G* polymorphism (Table 3). The *MTRR A66G* mutation was highest among 35–39 age group participants—27%. 

The second most prevalent was *MTHFR* gene polymorphism. From 4246 patients with preeclampsia tested for *MTHFR* gene mutations, 383 (9%) had confirmed *MTHFR C677T* polymorphism. This polymorphism was highest among the 30–34 age group women—26.63% (Table 3). 

The rarest type of polymorphism was the *MTR A2756G* mutation, with 88 cases (4.1%) confirmed among all patients with preeclampsia tested for an *MTR* polymorphism (N = 2140). Most of these cases were reported among patients of 30–34 and 35–39 age groups—25% and 22%, respectively. 

Results of chi-squared tests between age groups and types of genetic polymorphisms showed the prevalence of the *MTR A2756G* gene polymorphism conforms across different age groups, while there is a statistically significant association between age categories and the presence of the *MTHFR* gene polymorphism among the study population (*p* < 0.05). The prevalence of *MTRR* gene polymorphism also differs significantly across different age groups (Table 3). 

Results of chi-squared tests between polymorphisms revealed a statistically significant association between the *MTHFR C677T* gene polymorphism and the presence of the *MTRR A66G* gene polymorphism among the study population. There was also a statistically significant association found between the *MTR A2756G* gene polymorphism and the presence of the *MTRR A66G* gene polymorphism among the study population. However, no significant association between *MTHFR C677T* and the presence of *MTR* gene polymorphisms was found among the study population. 

**Figure 1:**
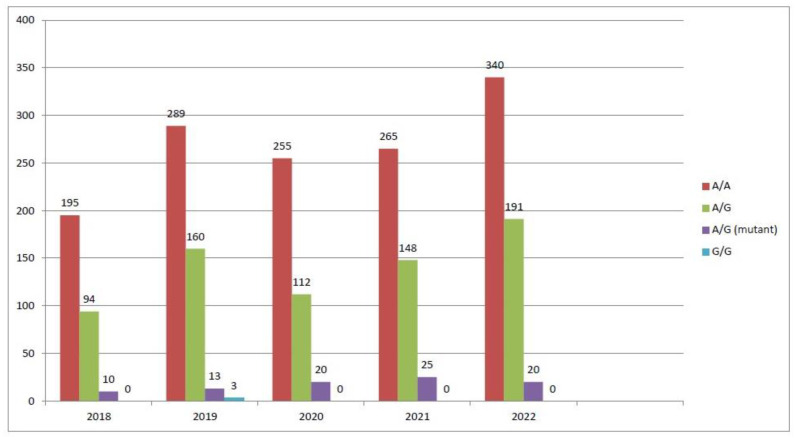
MTR gene polymorphism trends (2018–2022).

**Figure 2:**
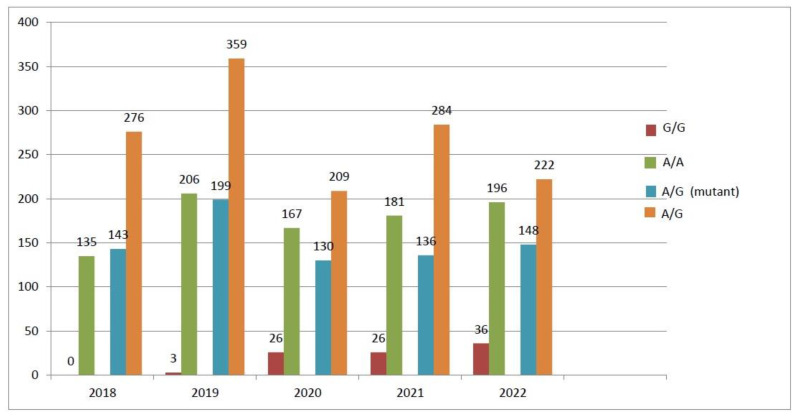
MTRR gene polymorphism trends (2018–2022).

**Figure 3:**
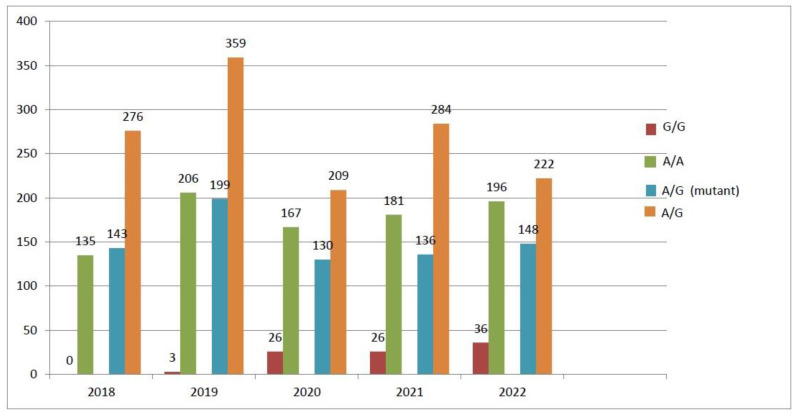
MTHFR gene polymorphism trends (2018–2022).

When the prevalence of specific gene polymorphisms was compared across the years for the period analyzed, the *MTR A2756G* polymorphism was slightly higher in 2020–2022 but not significantly (*p* > 0.05) (Figure 1), while *MTRR A66G* and *MTHFR C677T* polymorphisms were conformed over the study years (Figure 2 and Figure 3). 

Results of the HWE test show that our data deviate significantly from what would be expected under the assumptions of the Hardy–Weinberg equilibrium (*p*-value < 0.05). 

## 4. Discussion

Despite substantial improvements in the management [1,2], preeclampsia remains a huge health issue for many women during pregnancy resulting in adverse outcomes [6,7]. The role of thrombophilia and genetic polymorphism of specific genes responsible for coagulation homeostasis was proven [10,11,12,43], including the association between folate metabolism genes’ polymorphism in women with preeclampsia [25,26]. However, such studies have never been conducted among ethnic Kazakh women with preeclampsia, although there is data on increased *MTHFR* gene polymorphism among Kazakh ethnicity due to their specific diet [37]. Thus, this study’s aim was to evaluate the prevalence of folate metabolism genes’ polymorphisms *(MTR*, *MTRR,* and *MTHFR*) among ethnic Kazakh women with preeclampsia. 

The study results confirm the role and importance of folate genes’ polymorphisms in the development of preeclampsia. In this study, the highest prevalence of *MTR A2756G, MTRR A66G*, and *MTHFR C677T* polymorphism were registered among women with preeclampsia in the 30–34 and 35–39 age groups. This is in line with the local and international guidelines, which suggest that “maternal age 35 years or older” serves as a risk factor for preeclampsia [1]. In addition to the age-related risk, the appearance of the folate metabolism genes’ mutations contributes to the risk of preeclampsia. 

Significant expressions of *MTRR A66G* and *MTHFR C677T* were seen among the study participants and were related to preeclampsia. These findings are in agreement with previous studies investigating *MTRR A66G* and *MTHFR C677T* polymorphisms among women with preeclampsia of different ethnic and genetic backgrounds [17,20,25,26,46]. All these studies confirm the pathogenetic potential of these genes’ polymorphisms in the risk of preeclampsia development. Moreover, reviews and meta-analyses investigating the association between *MTHFR* and *MTRR* polymorphisms and preeclampsia confirm their pathogenetic role [18,21,22]. 

However, the *MTR A2756G* gene polymorphism was rarely seen in this report among women with preeclampsia. This finding is in concordance with the previous study investigating the association of *MTR A2756G* polymorphism and the development of preeclampsia [25,47]. These studies reported low association and unclear potential significance of *MTR A2756G* polymorphism in preeclampsia. However, our finding contradicts the report by Osunkalu et al. (2020), which confirmed *MTR A2756G* polymorphisms association with preeclampsia among Nigerian women [20]. The study from Chile also reported overexpression of *MTR* among women with preeclampsia [48], contrary to our findings. The researchers explain that the overexpression of *MTR* in the placenta of women with preeclampsia results in a “potential compensation mechanism” of folate metabolism in preeclampsia [49]. In general, limited reports are available on the role of *MTR A2756G* polymorphism in preeclampsia with inconsistent results. The contradiction of the results obtained in the discussed studies might be attributed to the differences in study design, confounding variables effect, and ethnic background of the participants. 

In this study, the prevalence of *MTR A2756G* polymorphism was higher in 2020–2022 compared to 2018 and 2019. This could be explained by the implementation of up-to-date and more efficient laboratory procedures and standardization of the workflow by the laboratory, which, in general, led to overall improvements in the diagnostics. Moreover, with the development of laboratory diagnosis in the country, genetic testing was made accessible to a wide cohort of patients, regardless of their socio-demographic status. 

In the context of this genetic study on folate metabolism genes’ polymorphisms (*MTR*, *MTRR*, and *MTHFR*) among ethnic Kazakh women with preeclampsia, an HWE test would evaluate whether the observed genotype frequencies match the expected frequencies based on the genetic principles. However, the genotype frequencies observed among the study participants do not align with what would be expected under the assumptions of the Hardy–Weinberg equilibrium. Several factors could lead to a significant deviation from HWE, including non-random mating, mutation (new mutations can introduce new alleles into the population, which can also disrupt equilibrium), patients’ selection, and genetic drift [50].

### Study Strengths and Limitations

To our knowledge, this is the first study investigating the prevalence of folate metabolism genes’ polymorphisms (*MTR*, *MTRR*, and *MTHFR*) among ethnic Kazakh women with preeclampsia in the Kazakhstani population. Moreover, a large number of participants over the 5-year period were included in the analysis. Nevertheless, some important limitations should be acknowledged. The retrospective nature of this study did not allow control patients to be included for comparison; however, it permitted the investigation at a reduced cost. Moreover, the laboratory electronic system did not provide detailed information on the study participants’ socio-demographic data (education, marital status, family income), past medical history, and past pregnancy history. The availability of these essential variables could enrich the study results and enable the evaluation of the influence of confounding risk factors. Moreover, the availability of information on the study participants’ homocysteine and folic acid levels could give an understanding of the relationship between the investigated genes’ mutations and the risk of preeclampsia. Thus, future investigations should be designed as a case-control study to compare folate gene polymorphisms in patients with preeclampsia and healthy controls and include evaluation of homocysteine and folic acid levels among the study participants. 

## 5. Conclusions

Although this study did not confirm the role of *MTR A2756G* in the development of preeclampsia, the high prevalence of *MTRR A66G* and *MTHFR C677T* polymorphisms were found among women aged 30–39 years old. These findings suggest the importance of evaluating *MTR*, *MTRR*, and *MTHFR* polymorphisms, especially the presence of all three *MTR A2756G*, *MTRR A66G*, and *MTHFR C677T* variants in women with preeclampsia.

## Figures and Tables

**Table 1:** The studied genes considered genetic risk factors for recurrent pregnancy loss.

Gene	Main Function	Polymorphism Name	Mutation	Pathologic Mechanism	Authors
*MTR*	*MTR* encodes the enzyme 5-methyltetrahydrofolate-homocysteine methyltransferase (interchangeable terminology-cobalamin-dependent methionine synthase), catalyzes the final step in methionine biosynthesis	*MTR A2756G*	The *A → G* polymorphism at position 2756 in the protein binding region of *MTR*, which replaces aspartic acid with glycine	Elevated homocysteine concentration	[21,43]
*MTRR*	The protein encoded by *MTRR* regenerates a functional methionine synthase via reductive methylation	*MTRR A66G*	*A66G* polymorphism (substitution of A for G at position 66) causes the substitution of isoleucine with methionine at codon 22 of *MTRR*	*MTRR* is responsible for the remethylation of homocysteine to methionine via a vitamin B12-dependent path	[21,23,25,43]
*MTHFR*	The protein encoded by this gene catalyzes the conversion of 5,10-methylenetetrahydrofolate to 5-methyltetrahydrofolate, a co-substrate for homocysteine remethylation to methionine	*MTHFR C677T*	*C677T* polymorphism is a point mutation at position 677 on the *MTHFR* gene with the substitution of cysteine to thymine nucleotide at that position, which causes the substitution of alanine to valine (Ala222Val) in the catalytic domain of *MTHFR*	Thermolabile variant the enzyme with reduced catalytic activity and deleterious effect on plasma homocysteine metabolism, leading to hyperhomocysteinemia and coagulation disorders	[10,21,25,36,43]

*MTR*—Methionine synthase; *MTRR*—methionine synthase reductase; *MTHFR*—methylenetetrahydrofolate reductase.

**Table 2:** Primer sequences and restriction enzymes utilized for detection of *MTR*, *MTRR*, and *MTHFR* polymorphisms.

Polymorphism	Temperature and Cycles	Restriction Enzyme	Products Size	Primer Sequences
*MTR A2756G*	53 °C, 36	*Nsp I*	151 ^a^	Forward: 5′-GAACTAGAAGACAGAAATTCTCTA-3′
*MTRR A66G*	60 °C, 35	*Hae III*	189 ^a^	Forward: 5′-GCAAAGGCCATCGCAGAAGACAT-3′
*MTHFR C677T*	58 °C, 35	*Hinf I*	198 ^a^	Forward: 5′-TGAAGGAGAAGGTGTCTGCGGGA-3′

^a^ RFLP product for the normal allele.

**Table 3:** Folate metabolism genes’ mutations among the study participants (2018–2022).

Variables	Genes (N = 4246)											
	*MTR, A2756G* Polymorphism, N = 2140 (100%)				*MTRR, A66G* Polymorphism, N = 3082 (100%) *				*MTHFR, C677T* Polymorphism, N = 4246 (100%) *			
Age groups	A/A	A/G	A/G (mutant)	G/G	G/G	A/A	A/G (mutant)	A/G	G/G	C/C	C/T	C/T (mutant)
mean, (SD±)	27.98 (12.05)	27.82 (11.92)	29.74 (11.49)	33.33 (1.53)	31.18 (5.13)	29.06 (10.57)	29.96 (11.14)	30.35 (29.74)	30.65 (5.2)	30.84 (24.32)	30.91 (27.48)	33.03 (52.53)
15–19	236 (65.19%)	126 (34.81%)	0	0	1 (0.25%)	114 (28.71%)	96 (24.18%)	186 (46.85%)	2 (0.45%)	216 (48.97%)	180 (40.81%)	43 (9.75%)
20–24	94 (65.28%)	44 (30.56%)	6 (4.17%)	0	7 (4.12%)	53 (31.18%)	36 (21.18%)	74 (43.53%)	24 (9.8%)	114 (46.53%)	83 (33.88%)	24 (9.8%)
25–29	258 (58.24%)	165 (37.25%)	20 (4.51%)	0	29 (4.63%)	176 (28.12%)	159 (25.4%)	262 (41.85%)	60 (6.9%)	410 (47.18%)	328 (37.74%)	71 (8.17%)
30–34	349 (65.85%)	156 (29.43%)	23 (4.34%)	2 (0.38%)	25 (2.96%)	278 (32.9%)	189 (22.37%)	353 (41.78%)	57 (4.73%)	614 (50.95)	432 (35.85%)	102 (8.46%)
35–39	273 (62.19%)	141 (32.12%)	24 (5.47%)	1 (0.23%)	26 (3.79%)	194 (28.28%)	172 (25.07%)	294 (42.86%)	50 (5.14%)	457 (46.97%)	377 (38.75%)	89 (9.15%)
40–44	91 (62.76%)	52 (35.86%)	2 (1.38%)	0	3 (1.18%)	49 (19.22%)	77 (30.2%)	126 (49.41%)	7 (1.93%)	171 (47.24%)	143 (39.5%)	41 (11.33%)
45–49	23 (60.53%)	14 (36.84%)	1 (2.63%)	0	0	14 (22.58%)	13 (20.97%)	35 (56.45%)	0	50 (56.18%)	33 (37.08%)	6 (6.74%)
50≤	20 (68.97%)	7 (24.14%)	2 (6.9%)	0	0	7 (17.07%)	14 (34.15%)	20 (48.78%)	0	23 (37.1%)	32 (51.61%)	7 (11.29%)
**Total**	**1344**	**705**	**88**	**3**	**91**	**885**	**756**	**1350**	**200**	**2055**	**1608**	**383**

*MTR*—Methionine synthase; *MTRR*—methionine synthase reductase; *MTHFR*—methylenetetrahydrofolate reductase. * χ^2^—*p* < 0.05.

## Data Availability

The dataset repository was created and available via the link https://zenodo.org/records/12724579 on the Zenodo repository, last accessed on 1 August 2024.
